# Ultrasensitive Detection of Porcine Epidemic Diarrhea Virus from Fecal Samples Using Functionalized Nanoparticles

**DOI:** 10.1371/journal.pone.0167325

**Published:** 2016-12-09

**Authors:** Na Xing, Xiaoxiao Guan, Bin An, Beibei Cui, Zengguo Wang, Xiaoya Wang, Xiujuan Zhang, Qian Du, Xiaomin Zhao, Yong Huang, Dewen Tong

**Affiliations:** College of Veterinary Medicine, Northwest A&F University, Yangling, Shaanxi, P. R. China; Sun Yat-Sen University, CHINA

## Abstract

Porcine epidemic diarrhea virus (PEDV) is the main causative agent of porcine diarrhea, which has resulted in devastating damage to swine industry and become a perplexed global problem. PEDV infection causes lesions and clinical symptoms, and infected pigs often succumb to severe dehydration. If there is not a timely and effective method to control its infection, PEDV will spread rapidly across the whole swine farm. Therefore, preclinical identification of PEDV is of great significance for preventing the outbreak and spread of this disease. In this study, a functionalized nanoparticles-based PCR method (UNDP-PCR) specific for PEDV was developed through systematic optimization of functionalized magnetic beads and gold nanoparticles which were further used to specifically enrich viral RNA from the lysate of PEDV stool samples, forming a MMPs-RNA-AuNPs complex. Then, oligonucleotides specific for PEDV coated on AuNPs were eluted from the complex and were further amplified and characterized by PCR. The detection limitation of the established UNDP-PCR method for PEDV was 25 copies in per gram PEDV stool samples, which is 400-fold more sensitive than conventional RT-PCR for stool samples. The UNDP-PCR for PEDV exhibited reliable reproducibility and high specificity, no cross-reaction was observed with other porcine viruses. In 153 preclinical fecal samples, the positive detection rate of UNDP-PCR specific for PEDV (30.72%) was much higher than that of conventional RT-PCR (5.88%) and SYBR Green real-time RT-PCR. In a word, this study provided a RNA extraction and transcription free, rapid and economical method for preclinical PEDV infection, which showed higher sensitivity, specificity and reproducibility, and exhibited application potency for evaluating viral loads of preclinical samples.

## Introduction

With the development of modern intensive swine-raising industry, there is a dramatic increase in the number of pigs infected by enteropathogenic viruses, such as porcine epidemic diarrhea virus (PEDV), transmissible gastroenteritis virus (TGEV) and porcine rotavirus (PoRV) [1–5]. Among these viruses, the positive rate of PEDV is relatively higher in the whole world and it causes considerable economic losses to pig-production in recent years [6]. PEDV is the major causative agent of porcine epidemic diarrhea, which was first discovered in the United Kingdom in 1971, afterwards this pathogen spread throughout European and Asian countries, such as the Belgium, United States, Japan, Korean, China and Vietnam, resulting in serious damage to pig producers [7–12]. This disease is clinically characterized by vomiting, dehydration and severe watery diarrhea. Pigs of any age could be infected by PEDV, from newborn pigs to boars or sows. In sucking piglets, the morality rate can reach 80%-100% [6].

PED is indistinguishable from other porcine diarrheal diseases including TGE and rotavirus diarrhea based on clinical symptoms and necropsy. Therefore, it is necessary to definitely identify the causative pathogen using confirmatory laboratory tests, which is essential for timely clinical decision-making and management of epidemics of PED [13]. Commonly used laboratory diagnostic methods of PEDV include antigen enzyme-linked immunosorbent assay (ELISA), immunochromatography assay (IC), reverse transcriptase polymerase chain reaction (RT-PCR), reverse transcription loop-mediated isothermal amplification (RT-LAMP), TaqMan-based real-time RT-PCR and nanoparticle-assisted PCR assay and so forth [14–20]. Currently, ELISA and IC have been widely applied to detect PEDV in large-scale blood or feces samples. In these two methods, a highly specific monoclonal antibody targeted to one viral epitope needs to be designed and produced, so they are not able to detect some of the PEDV strains when lacking specific detection antibody. Additionally, in initial stage of PEDV infection the virus titer is relatively low, so ELISA and IC sometimes may not detect the presence of PEDV. Nucleic acids based detection approaches (RT-LAMP and real-time RT-PCR) gain more sensitivity than antigen-based methods (ELISA and IC), but they also have some shortcomings which hinders its wide application. For example, RT-LAMP has a strict demand for designing specific primers and the identification of LAMP products is not easy because of products contamination. In addition, real-time RT-PCR requires special instruments and dye-labeled probes, making them unsuitable in clinical practice. Nanoparticle-assisted PCR assay is an advanced form of PCR in which nanoparticles are used to increase thermal conductivity, and the sensitivity of this method is 100-fold that of conventional RT-PCR [20]. However, existing established PCR-based assays of detecting PEDV need RNA extraction, purification and reverse transcription of RNA, which are time-consuming and laborious. Moreover, during this complicated process, RNA is more likely to be degraded by RNAase in the environment. Therefore, it is urgent to develop a rapid, highly sensitive and economical method, allowing for point-of-care (POC) detection of PEDV.

Nanoparticles have lots of nanometer characteristics, such as multivalency, superparamagnetism, modified functional chemical groups (such as -COOH, -SH, -NH_2_) on surface [21–23]. Recently, nanoparticles in different sizes, materials and shapes have been increasingly applied in developing biosensors for detecting pathogens and corresponding antibodies or specific biomarkers on cells. In our study, a functionalized nanoparticles-based PCR method (UNDP-PCR) was developed to identify PEDV infection in preclinical stool samples, in which magnetic microparticles and gold nanoparticles are employed. PEDV, as one of the members of family *Coronaviridae*, is an enveloped virus with a positive-sense, single-stranded RNA genome of 28kb. From 5’ cap to the 3’ poly A tail. PEDV genomic RNA consists of seven open reading frames (ORFs), encoding viral replicase, spike protein (S), ORF3, envelope protein (E), membrane protein (M), nucleocapsid protein (N) in sequence. Unlike other proteins, ORF1a gene encoding viral replicase 1a only exists in genomic RNA (gRNA) and has low-level genetic mutations [24, 25]. Therefore, PEDV specific probes and oligonucleotides were designed on the basis of the conserved region of ORF1a, which could quantify infectious viral particles accurately. Via systematical optimization, magnetic microparticles (MMPs) coated with PEDV specific probes and gold nanoparticles (AuNPs) coated with PEDV specific oligos were used to enrich viral genomic RNA and amplify weak viral signals from fecal samples. Then MMPs-RNA-AuNPs complexes were formed, plenty of DNA barcodes on the surface of gold nanoparticles were released and characterized by PCR. Therefore, the established UNDP-PCR assay for PEDV is fit for diagnosis of PEDV in early stage of viral infection, possessing higher sensitivity, specificity and reproducibility.

## Materials and Methods

### Ethics statement

Approval of the study was obtained on May 20th 2015 from the Ethical Committee for Animal Experiments of the Northwest A&F University.

### Virus strains and cells

PEDV strain (GenBank No. AF353511), PRRSV Shaanxi strain (GenBank No. HQ401282), TGEV strain (GenBank No. HQ462571), PCV2 strain (GenBank No. EU366323) and PPV YL strain (GenBank No. JN860197) used in this study were isolated and purified previously by our research team [26–30]. The CSFV Shimen strain (GenBank No. AY775178) was kindly provided by Prof. Yanming Zhang (College of Veterinary Medicine, Northwest A&F University) [31]. In this study, these virus strains were used as standard viruses and maintained at -80°C until used. PEDV was propagated in Vero cells supplemented with exogenous trypsin. PRRSV was propagated in Mac-145 cells. TGEV, PCV2 and PPV were propagated in PK-15 cells. CSFV was propagated in ST cells. The four kinds of cells including Vero, Mac-145, PK-15 and ST were cultured in Dulbecco’s modified eagle medium (DMEM) (Gibco, Gaithersburg, MD, USA) supplemented with 10% heat inactivated fetal bovine serum (FBS).

### Field samples

During the period from November 2015 to January 2016, a total of 153 fecal samples were collected from apparently healthy pigs in three different pig-raising farms (Qinliangshan, Lvjianyuan and Fuyuan) near Yangling located in Huxian county, Xi’an, Shaanxi province, China. The Qinliangshan pig farm is located in the south of Zhenfucunyaocun, Jiang Village (34°2'7'' north latitude and 108°31'47'' east longitude). Lvjianyuan and Fuyuan pig farms are located in the east of Lijiazhuangcun, Yuchan Village (34°6'35'' north latitude and 108°33'32'' east longitude) and Sunjiawei cun, Yuchan Village (34°6'27'' north latitude and 108°34'38'' east longitude) respectively. Fresh fecal samples were collected from individual pigs on farms, placed in sterile specimen cups, kept on ice, and returned to the laboratory within 12 h. Then stool samples (weight: about 1g) were diluted in PBS buffer, followed by vortex and centrifugation at 4,000 × g for 10 min to eliminate fecal debris. Then the supernatants of per gram stool samples were tested using conventional RT-PCR, SYBR Green real-time PCR and UNDP-PCR for PEDV, respectively. In this experiment, we have obtained the permission from the three pig-rearing farms mentioned above to collect pig fecal samples.

### Extraction and reverse transcription of viral nucleic acids

Viral RNA/DNA Kits (OMEGA, USA) were used to extract different viral nucleic acid (RNA and DNA) from culture supernatants of different viruses or fecal samples. The extraction procedure was performed according to manufacturer’s instructions. Then the RNA extracted from RNA viruses were reversely transcripted into complementary DNA (cDNA) using the reverse transcriptase kit (Takara Corp., Japan) according to manufacturer’s protocols.

### Primers design and probes selection

In UNDP-PCR, probes and oligonucleotides specific for PEDV were designed by comparing multiple sequences of complete ORF1a gene (about 12.3kb) of 326 PEDV strains in Genbank using DNASTAR and VECTOR NTI software packages. Highly conserved region of PEDV ORF1a with the size of more than 25 bp are mainly situated in the following regions (CV777): 71–105, 211–242, 622–652, 2095–2122, 2179–2204, 2543–2571, 2752–2778, 2890–2915, 3274–3315, 3406–3437, 3449–3485, 3762–3793, 4132–4193, 4243–4272, 4274–4311, 4614–4660, 4959–4995, 5061–5093, 5200–5234, 5249–5288, 5338–5372, 5392–5423, 5878–5917, 6098–6131, 6221–6260, 6371–6398, 6682–6719, 6779–6815, 6867–6894, 7006–7031, 7046–7082, 7084–7113, 7144–7194, 7324–7352, 7379–7412, 7522–7550, 7858–7892, 7896–7934, 7936–7979, 8068–8106, 8255–8283, 8494–8528, 8560–8585, 8662–8696, 8775–8801, 8839–8870, 9103–9133, 9232–9263, 9481–9512, 9760–9787, 10198–10229, 10603–10636, 10684–10716, 10859–10931, 10974–11006, 11059–11105, 11353–11381, 11496–11541, 11659–11696, 12037–12098, 12109–12137, 12145–12182, 12202–12248. In consideration of the binding stability, difficulty degree of hybridization and mismatch rates, the designed PEDV specific primers used in UNDP-PCR assay do not contain more than three continuous nucleotides (G/C/A/T) and their GC content is over 40%,. In addition, because of steric hinerance and capture efficiency, the distance of every pair of primers is between 500–600 bp. Relying on these selecting principles, 8 pairs of PEDV specific primers were designed (16 targeted sequence sites in total). In addition, PEDV specific primers used in conventional RT-PCR detection method were designed using Primer Premier and Primer-blast software according to PEDV highly conserved sequences in replicase protein-encoding region ORF1a. Previously published primers for conventional PCR/RT-PCR method of detecting PRRSV, TGEV, PCV2, CSFV and PPV were used in the present study [32, 33]. All the probes, oligos and primers presented in Tables 1 and 2 were specific to the targeting gene sequences and synthesized by Sangon (Shanghai, China) and Genscript (Nanjing, China).

**Table 1:** Primers and selected probes used in this study.

Label	Orientation	Assay	Sequence (5′–3′)	Position
pGL3-F	Forward	LINK-PCR	TGGCATTCCGGTACTGTTGG	58–77
pGL3-R	Reverse	LINK-PCR	AGGAGAATAGGGTTGGCACC	967–948
PEDVF	Forward	Conventional-PCR	GTGGTAACATCGTGCCAGT	618–636
PEDVR	Reverse	Conventional-PCR	GTCCAATCACCAACAGTCC	1050–1068
PEDV-DP1	Forward	Detect-PCR	GATGCACATATCGAGGTGG	
PEDV-DP2	Reverse	Detect-PCR	AAGTGTCCACATACGCAC	
Probe 1		Hybridization	5’NH_2_-T_15_GCCTCAGAATAGTATGAGACGGCTTC	76–101
Probe 2		Hybridization	5’NH_2_-T_15_CCTGTAGATAGTAGCAAGTGGCTCAG	626–651
Probe 3		Hybridization	5’NH_2_-T_15_GTTCCTGGTACCCATAGAGACAGACC	6234–6259
Probe 4		Hybridization	5’NH_2_-T_15_ATAGTGGCAGGACCTGTCGGTTGCAC	6784–6809
Probe 5		Hybridization	5’NH_2_-T_15_AGACAAACTGGCCAACAACGCTGAGT	7046–7071
Probe 6		Hybridization	5’NH_2_-T_15_GCACCCTTCTTATTTGCAATGCAAAC	7525–7550
Probe 7		Hybridization	5’NH_2_-T_15_TTGGCACCTACACGCATACACGTGGC	7324–7349
Probe 8		Hybridization	5’NH_2_-T_15_TTACCAGCTAAATAAACACCTGCTGG	7858–7883
Probe 9		Hybridization	5’NH_2_-T_15_AGCTACAAGCCACACCACAGGTATAC	7382–7407
Probe 10		Hybridization	5’NH_2_-T_15_CCCTTATCAGCAACGCCACTAGCATC	7936–7961
Probe 11		Hybridization	5’NH_2_-T_15_TGTGCCACAAACAAAGTCAGAACCAG	8258–8283
Probe 12		Hybridization	5’NH_2_-T_15_TAGAGTTGGCAAGTCTCTCATAGGCA	8775–8800
Probe 13		Hybridization	5’NH_2_-T_15_CCTTTATCTGCAACAAAGCACCTCGC	9234–9259
Probe 14		Hybridization	5’NH_2_-T_15_GGATTGAGGCCAACAAAGCTTGTACA	9762–9787
Probe 15		Hybridization	5’NH_2_-T_15_AGTAGATGCAACACTCTGCAACATAC	10976–11001
Probe 16		Hybridization	5’NH_2_-T_15_ACTCAGCATTCTGTGCGGTTACCTCC	11496–11521

**Table 2:** Selected oligos used in this study.

Label	Orientation	Assay	Sequence (5′–3′)	Position
Oligo 1		Hybridization/Amplification	5’SH-T_15_GCCTCAGAATAGTATGAGACGGCTTCACCGTGATGGAATGGA	76–101
Oligo 2		Hybridization/Amplification	5’SH-T_15_CCTGTAGATAGTAGCAAGTGGCTCAGACCGTGATGGAATGGA	626–651
Oligo 3		Hybridization/Amplification	5’SH-T_15_GTTCCTGGTACCCATAGAGACAGACCACCGTGATGGAATGGA	6234–6259
Oligo 4		Hybridization/Amplification	5’SH-T_15_ATAGTGGCAGGACCTGTCGGTTGCACACCGTGATGGAATGGA	6784–6809
Oligo 5		Hybridization/Amplification	5’SH-T_15_AGACAAACTGGCCAACAACGCTGAGTACCGTGATGGAATGGA	7046–7071
Oligo 6		Hybridization/Amplification	5’SH-T_15_GCACCCTTCTTATTTGCAATGCAAACACCGTGATGGAATGGA	7525–7550
Oligo 7		Hybridization/Amplification	5’SH-T_15_TTGGCACCTACACGCATACACGTGGCACCGTGATGGAATGGA	7324–7349
Oligo 8		Hybridization/Amplification	5’SH-T_15_TTACCAGCTAAATAAACACCTGCTGGACCGTGATGGAATGGA	7858–7883
Oligo 9		Hybridization/Amplification	5’SH-T_15_AGCTACAAGCCACACCACAGGTATACACCGTGATGGAATGGA	7382–7407
Oligo 10		Hybridization/Amplification	5’SH-T_15_CCCTTATCAGCAACGCCACTAGCATCACCGTGATGGAATGGA	7936–7961
Oligo 11		Hybridization/Amplification	5’SH-T_15_TGTGCCACAAACAAAGTCAGAACCAGACCGTGATGGAATGGA	8258–8283
Oligo 12		Hybridization/Amplification	5’SH-T_15_TAGAGTTGGCAAGTCTCTCATAGGCAACCGTGATGGAATGGA	8775–8800
Oligo 13		Hybridization/Amplification	5’SH-T_15_CCTTTATCTGCAACAAAGCACCTCGCACCGTGATGGAATGGA	9234–9259
Oligo 14		Hybridization/Amplification	5’SH-T_15_GGATTGAGGCCAACAAAGCTTGTACAACCGTGATGGAATGGA	9762–9787
Oligo 15		Hybridization/Amplification	5’SH-T_15_AGTAGATGCAACACTCTGCAACATACACCGTGATGGAATGGA	10976–11001
Oligo 16		Hybridization/Amplification	5’SH-T_15_ACTCAGCATTCTGTGCGGTTACCTCCACCGTGATGGAATGGA	11496–11521

### Preparation of PEDV specific probe-coated MMPs

First, carboxylated-modified magnetic beads (MyOne Dynabeads; Invitrogen, Inc., Carlsbad, CA, USA) were suspended in activated buffers (MES). Then, EDC and NHS (Sigma-Aldrich, St. Louis, MO, USA) were added and used for activation of magnetic beads. Subsequently, 5’ amino (NH_2_)-modified PEDV specific probes were added to the activated magnetic beads solution, establishing a peptide bond between them during incubation for more than three hours at room temperature. Finally, MMPs-probes were separated using a magnetic scaffold, then resuspended in TE solution and stored at 4°C until further use [21, 34].

### Preparation of PEDV specific oligo-coated AuNPs

1 ml of gold nanoparticles (AuNPs) with diameter of 15nm was centrifuged at 7,500 g for 30 minutes. After discarding the supernatants, the AuNPs were resuspended in 100 μl deionized water. After that, the resuspended AuNPs and PEDV specific oligonucleotides modified with 5’ sulfydryl (SH) were mixed together, and incubated for more than 48 hours at room temperature with gentle shaking to form covalent Au-S bond by slow salt to a final concentration of 0.01 M PBS (0.1 M NaCl in 0.01 M of phosphate buffer, PH 7.0). Unbound thiolated PEDV specific oligonucleotides were removed by centrifugation and rinsing with PBS buffer. Then, the functionalized AuNPs were resuspended in 0.01 M PBS and stored at 4°C until used.

### Procedure of UNDP-PCR for detection of PEDV

Collected fecal samples were diluted in PBS buffer, then vortexed, followed by centrifugation at 4,000 g for 10 minutes to eliminate fecal debris. Then the fecal supernatants mixed with equal volume of lysis buffer were boiled for 15 minutes to release PEDV genomic RNA. The boiled products were transferred to one hybridization tube containing 2 μl of MMPs coated with PEDV specific probes and 20 × hybridization buffer (20×SSC, 1% Tween-20 and 2% SDS in H_2_O). These components were mixed by vortexing, followed by incubation at 40°C for 30 minutes. Then, 2 μl AuNPs coated with PEDV specific oligo were added to the hybridization system and incubated at 50°C for 40 minutes by stirring. The MMPs-RNA-AuNPs complexes were washed twice with 1 mL TE buffer and separated magnetically to remove unbound functionalized AuNPs and residual hybridization buffer.

Elution buffer (0.5 M DTT, 10 mM Tris-HCl, 1 mM EDTA, pH 7.5) was used for release of thiolated DNA barcodes from the surface of functionalized AuNPs. The eluted DNA barcodes were purified and precipitated with NaAc and absolute alcohol, then were mixed with specific capture single strand DNA (ssDNA), followed by conventional PCR assay using PEDV specific detect-PCR primers (Table 1) as described in the previous study [33, 35]. The amplification products were separated on 1.5% agarose gel stained with ethidium bromide (EB) and visualized under ultraviolet (UV) light.

### The specificity, sensitivity and reproducibility of the UNDP-PCR assay for PEDV

Purified PEDV genomic RNA was subjected to reverse transcription into cDNA with random primers. Then, partial genome sequence of PEDV ORF1a was amplified from synthesized cDNA using the specific primers (Forward: 5’-GTGGTAACATGCCAGT-3’ Reverse: 5’-GTCCAATCACCAACAGTCC-3’). The PCR products with the size of 451 bp were purified using gel extraction kit and cloned into pMD19-T vector (TAKARA, Japan) to construct the standard plasmid. Then the constructed plasmid was sequenced by Genscript (Nanjing, China).The plasmid concentration was determined by Nanodrop 2000 Spectrophotometer (Thermo scientific, USA), then the plasmid copy number was calculated as described in the previous study [33, 35]. To test the specificity of the UNDP-PCR for PEDV, other viruses including PRRSV, TGEV, PCV2, PPV and CSFV were added to the reaction system and tested. To test the sensitivity, serially diluted samples with the range from 10^5^ to 1 copies per gram were detected by conventional RT-PCR and UNDP-PCR. The inter-assay and intra-assay was carried out in triplicates of each concentration (10^4^ copies/ gram, 10^3^ copies/ gram, 10^2^ copies/ gram) by three independent tests for three consecutive days to test the reproducibility of this assay.

## Results

### Probes design and optimization of UNDP-PCR assay for detecting PEDV

**Fig 1:**
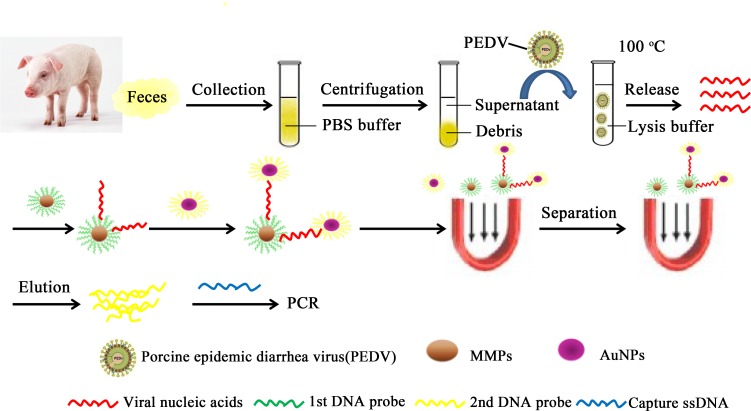
Schematic of detecting PEDV from fecal samples using functionalized nanoparticles.

To find an economical and rapid diagnostic method with higher specificity, sensitivity and reproducibility for PEDV preclinical infection, relevant experiments were implemented to establish a systematic and optimal protocol for UNDP-PCR assay for PEDV, as schematically depicted in Fig 1.

In our previous study, we have optimized a UNDP-PCR assay for TGEV in which the probes and oligos were targeted to replicase protein-encoding region ORF1a [35]. Quite similar to TGEV in genetic structure, the 5’ two-thirds of PEDV genome encodes two polyproteins (1a and 1b) necessary for RNA replication and only exists in genomic RNA (gRNA). However, the 3’ one-third encodes nonstructural protein (ORF3) and structural proteins, including spike (S), envelope (E), membrane (M) and nucleocapsid (N) proteins, existing in both genomic RNAs (gRNAs) and subgenomic mRNAs (sgmRNAs) [36]. The results showed that viral numbers quantified by real-time PCR assay were significantly different using specific primers targeted to gRNAs and different sgmRNAs. Thus, specific probes and oligos in UNDP-PCR assay for PEDV were designed based on ORF1a region, which could truly reflect the level of viral infection. To ensure wide detection range of this UNDP-PCR assay for PEDV, 16 highly conserved sequences in ORF1a region from all PEDV strains published on National Center of Biotechnology Information (NCBI) were selected to design PEDV specific primers used in UNDP-PCR.

**Fig 2:**
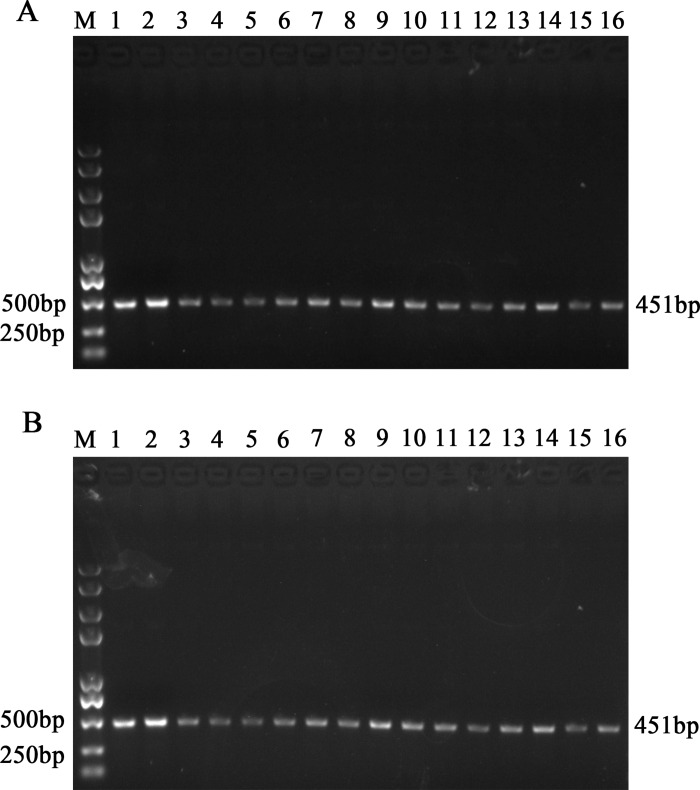
Comparison of different functionalized MMPs and AuNPs. (A) Probes-coated magnetic microparticles MMPs-p1 to p16 were incubated with RNA of PEDV in hybridization buffer at 40°C for 30 minutes, followed by rinsing and magnetic separation. Then the MMPs-RNA complexes were subjected to PEDV-specific RT-PCR. M: Trans 2K Plus DNA Marker; 1: p1; 2: p2; 3: p3; 4: p4; 5: p5; 6: p6; 7: p7; 8: p8; 9: p9; 10: p10; 11: p11; 12: p12; 13: p13; 14: p14; 15: p15; 16: p16. (B) PEDV RNA was incubated with oligo1 to 16 coated Au-NPs at 50°C for 40 minuts. Then the complexes were washed and precipitated by centrifugation, followed by reverse transcription and PEDV specific RT-PCR detection. M: Trans 2K Plus DNA Marker; 1: oligo1; 2: oligo2; 3:oligo3; 4: oligo4; 5: oligo5; 6: oligo6; 7: oligo7; 8: oligo8; 9: oligo9; 10: oligo10; 11: oligo11; 12: oligo12; 13: oligo13; 14: oligo14; 15: oligo15; 16: oligo16.

Firstly, the magnetic microparticles coated with designed probes 1–16 were prepared and were named MMPs-p1 to p16. To select and determine optimal probes for capture of PEDV genomic RNA, functionalized MMPs-p1 to p16 were incubated with PEDV RNA in hybridization buffer at 40°C for 30 minutes to form MMPs-RNA complexes, followed by reverse transcription and specific conventional PCR detection to determine capture efficiency of these MMPs-probes. The experimental results indicated that all 16 designed probes were capable of capturing RNA of PEDV. Among them, MMP-p1 and MMP-p2 showed higher efficiency in capturing PEDV genome (Fig 2A). Next, SH-modified oligonucleotides (oligos) 1–16 sharing same nucleic acid sequences of probe 1–16 were coated to gold nanoparticles (AuNPs) to prepare AuNPs-oligo1 to 16. Then PEDV RNA was incubated with oligo-functionalized AuNPs at 50°C for 40 minutes. The formed complexes were then centrifuged and detected by PEDV specific RT-PCR. The results showed that among these designed oligos, oligo1 and oligo 2 also appeared higher binding efficiency with PEDV genomic RNA (Fig 2B). Based on above results, probe 1- functionalized MMPs and oligo 2-functionalized AuNPs are optimal for capturing nucleic acids of PEDV and formation of MMPs-RNA-AuNPs sandwich-like complexes.

### The sensitivity and reproducibility of UNDP-PCR assay for PEDV

**Fig 3:**
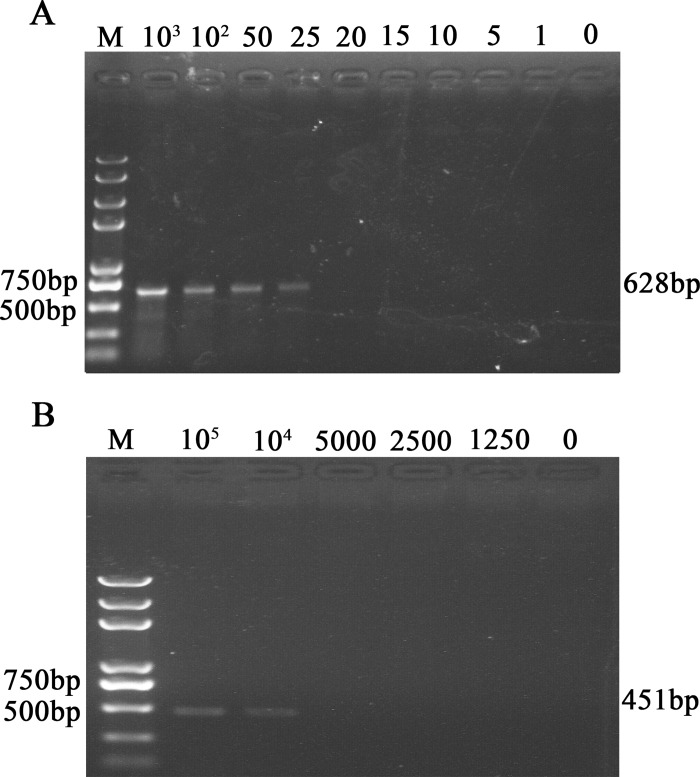
Analysis of the sensitivity of functionalized nanoparticles-based PCR assay for PEDV. (A) Serial dilutions of PEDV fecal samples were detected by functionalized nanoparticles-based PCR assay for PEDV. (B) Serial dilutions of PEDV fecal samples were detected by conventional RT-PCR.

Samples of purified PEDV were diluted serially and were used to test the sensitivity of this UNDP-PCR assay. Briefly, serial dilutions were boiled with lysis buffer containing RNAase inhibitors to release viral RNA, after which prepared functionalized MMPs and AuNPs were added to the reaction tube to form complexes with PEDV RNA. Then, MMPs-RNA-AuNPs complexes were washed and magnetically separated, followed by barcodes elution, purification and detection by PCR. The amplified products were analyzed using agarose gel electrophoresis and visualized by ethidium bromide staining under UV lights. As shown in Fig 3A, visible bands around 628 bp could be seen in lanes with viral concentrations ranging from 10^3^ to 25 copies per gram, suggesting the detection limit of UNDP-PCR for PEDV was about 25 copies/g. However, conventional RT-PCR was able to detect over 10^4^ copies of PEDV in 1 gram of stool samples (Fig 3B), indicating that the sensitivity of UNDP-PCR specific for PEDV was 400-fold that of the conventional RT-PCR.

**Fig 4:**
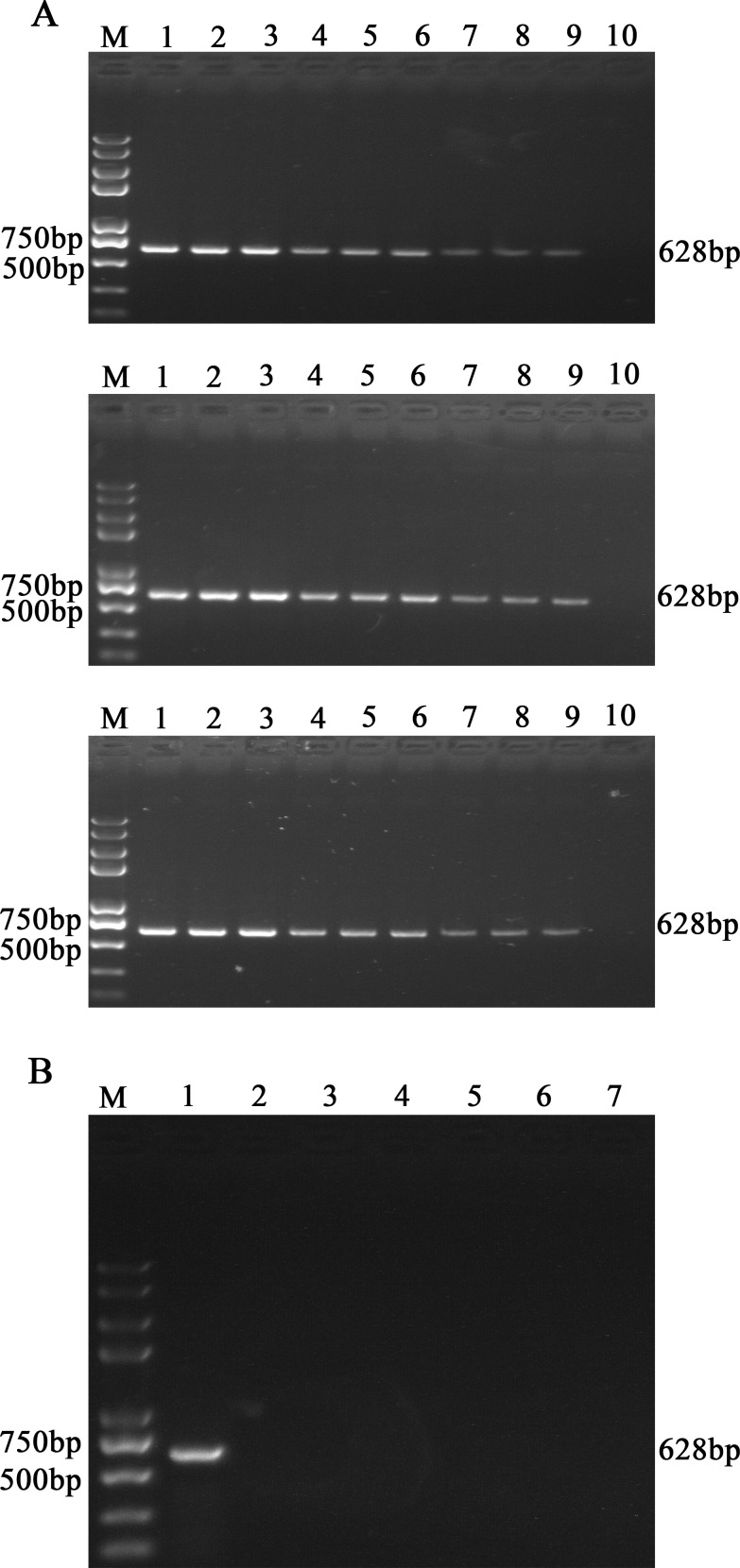
Analysis of the reproducibility and specificity of functionalized nanoparticles-based PCR assay for PEDV. (A) Three different concentrations of PEDV samples were detected by functionalized nanoparticles-based PCR assay for PEDV in triplicates and in three independent runs. Lane M: Trans 2K Plus DNA Marker; lane 1–3: 10^4^; lane 4–6: 10^3^; lane 7–9: 10^2^; lane10: negative samples. (B) PCV2, PPV, TGEV, CSFV, PRRSV and stools collected from healthy swine were detected by functionalized nanoparticles-based PCR assay for PEDV as control. Lane M: Trans 2K Plus DNA Marker; lane 1: PEDV; lane 2: the stools of healthy swine; lane 3: PCV2; lane 4: PPV; lane 5: TGEV; lane 6: CSFV; lane 7: PRRSV.

To evaluate the reproducibility of our established detection method, inter- and intra-assay test was carried out as followed. Three different concentrations of PEDV samples were selected: 10^4^ copies/g, 10^3^ copies/g and 10^2^ copies/g. Then, three replicates of each concentration were tested in three independent runs for three continuous days. As our prediction, the results of the independent triplicates assay showed highly consistency (Fig 4A).

### The specificity of UNDP-PCR assay for PEDV

The specificity of the UNDP-PCR assay for PEDV was evaluated using fecal samples collected from healthy pigs and other porcine viruses including TGEV, PCV2, PRRSV, CSFV and PPV. The analysis of agarose gel electrophoresis showed that this established detection method only exhibited a positive reaction with PEDV. Specific amplified products with the expected size of 628 bp were not obtained in the presence of other five pathogens (TGEV, PCV2, PRRSV, CSFV and PPV) and samples of healthy pigs. These results indicated that UNDP-PCR for PEDV did not have cross-reaction with other viruses, owning high specificity for PEDV (Fig 4B).

### Application of UNDP-PCR assay for PEDV pre-clinical infection

**Fig 5:**
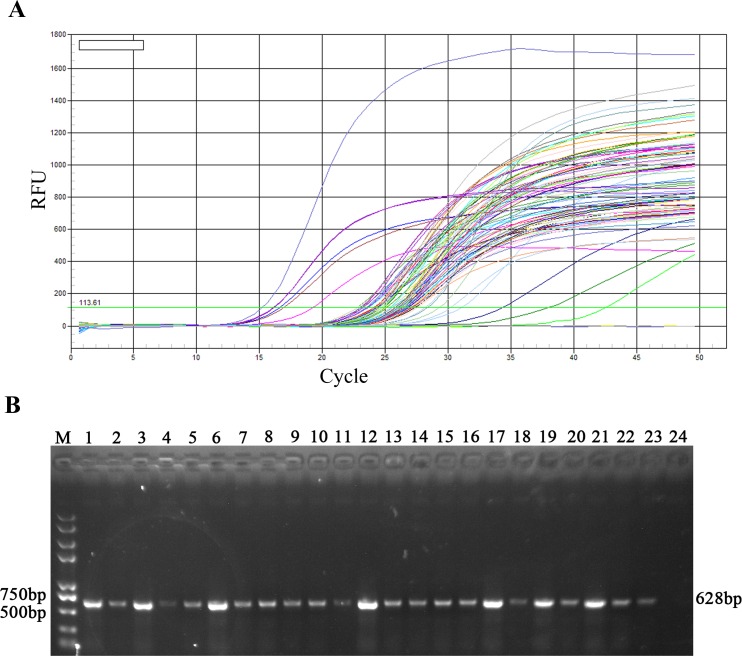
Detection of preclinical samples using real-time RT-PCR and functionalized nanoparticles-based PCR assay for PEDV. (A) SYBR Green real-time RT-PCR assay detected the all of positive samples and part negative samples identified by functionalized nanoparticles-based PCR assay. The figure showed all of positive samples and part negative samples identified by real-time RT-PCR for PEDV. (B) Agarose gel electrophoresis analysis of the relative viral load levels of 20 selected PEDV positive preclinical fecal samples identified by functionalized nanoparticles-based PCR assay specific for PEDV. Lane M: Trans 2K Plus DNA Marker; lane 1–20: preclinical fecal specimens; lane 21: 10^4^ Standards; lane 22: 10^3^ Standards; lane 23: 10^2^ Standards; lane 24: negative samples.

A total of 153 fecal samples collected from neighbouring pig farms were tested by conventional RT-PCR, SYBR Green real-time RT-PCR and UNDP-PCR specific for PEDV. As shown in Table 3, among 153 samples, 47 samples were positive by UNDP-PCR, 9 samples were found to be positive by conventional RT-PCR and 27 samples were positive by real-time RT-PCR (Fig 5A). Therefore, the PEDV positive detection rate of UNDP-PCR, conventional RT-PCR and real-time RT-PCR were 30.72%, 5.88% and 17.65% respectively. Next, to determine the viral loads in PEDV positive fecal samples, relative band intensities of 20 samples selected from 47 PEDV positive samples were compared with that of standard PEDV samples (10^4^, 10^3^, 10^2^ copies per gram). As shown in Fig 5B, among the 20 PEDV positive samples, 4 sample was over 10^4^ copies/g, 4 samples were between 10^4^ and 10^3^ copies/g, 10 samples were between 10^3^ and 10^2^ copies/g, and 2 samples were below 10^2^ copies/g.

**Table 3:** Comparison of the detection rate of PEDV infected preclinical fecal samples by conventional RT-PCR, functionalized nanoparticles-based PCR and SYBR Green real-time RT-PCR assay.

	Conventional RT-PCR	Functionalized nanoparticles-based PCR	SYBR Green real-time RT-PCR
Total number of tested samples	153	153	153
Number of positive samples	9	47	27
Number of negative samples	144	106	126
Positive rate (%)	5.88	30.72	17.65

## Discussion

Although inactivated and attenuated vaccines against PEDV are used in some pig-raising areas, PEDV infection still poses a huge threat to swine industry [12]. Up to now, the surveillance of PEDV is mainly dependent on identification in early stage of viral infection to prevent the spread of the infectious pathogen among pigs.

In this study, a UNDP-PCR assay for PEDV was successfully developed by adopting technology advances in nanotechnology and optimized by selecting specific probes for enrichment of viral nucleic acids. Therefore, this method gains more merits than existing laboratory detection methods of PEDV. Firstly, the developed PEDV UNDP-PCR assay has higher sensitivity. Accordingly, functionalized magnetic beads and nanoparticles were used to facilitate binding between viral nucleic acids and PEDV specific probes. Then, large number of DNA barcodes carried by probe-coated AuNPs were diluted and detected as templates for PCR. Obviously, the weak viral signals in small amount of fecal samples were highly amplified, resulting in that 25 viral copies of PEDV in per gram fecal samples could be detected. However, PEDV infection could only be detected by conventional RT-PCR methods when virus titer is over 10^4^ copies per gram fecal samples. Obviously, the functionalized nanoparticles-based PCR detection method for PEDV is approximately 400-fold more sensitive than that of conventional RT-PCR. Therefore, PEDV infection could be timely monitored by this method, especially in early stage of infection, which would be helpful for preventing virus particles from spreading to other pig farms. Secondly, to determine specificity of this functionalized nanoparticles-based detection method, other viruses such as TGEV, PCV2, CSFV, PPV and PRRSV which are likely to cause co-infection in pigs were examined. No amplification was obtained from these viruses, suggesting that this method has a high level of specificity for PEDV. To ensure diagnosis precision, specific DNA probes bound to magnetic microparticles and gold nanoparticles are designed targeting two distinct highly conserved sequences in viral ORF1a region, and then were selected through systematical optimization according to specific selection principles, such as capture efficiency, steric hinerance effect and so on. In addition, unlike commonly used commercial ELISA kits for PEDV, almost all PEDV strains could be detected by this assay, due to the fact that specific probes used to form sandwich-like complex with viral nucleic acids are directed against highly conserved region of PEDV ORF1a. In addition, large-scale preclinical stool samples could be detected simultaneously without the need for RNA extraction and reverse transcription in the same reaction system, so the whole detection process of UNDP-PCR for PEDV could be completed in a short period of time, and provide the viral loads in PEDV positive fecal samples. Therefore, this developed assay saves time, money and labor, showing a wide detection range and the potential for clinical application. However, as a new method, the optimization of reaction system and condition is a long and complicated process. Therefore, how to make this method more convenient will be the next thing we need to do.

## Conclusion

This optimized functionalized nanoparticles-based diagnosis method for PEDV possessed high-level sensitivity, specificity and reproducibility, providing a reliable platform for epidemiological investigation and monitoring of PEDV. This assay could increase detection rate of PEDV infection and is useful for evaluating the level of viral infection in large-scale pre-clinical fecal samples. In general, this assay developed in our study is rapid, ultrasensitive and cost-effective, which is able to control the occurrence, spread and massive outbreaks of porcine epidemic diarrhea effectively.
